# Harnessing brain activity at adolescence prevents later schizophrenia development

**DOI:** 10.1111/cns.13126

**Published:** 2019-04-02

**Authors:** Dong‐Sheng Zhou, Ti‐Fei Yuan

**Affiliations:** ^1^ Ningbo Kangning Hospital Ningbo China; ^2^ Shanghai Key Laboratory of Psychotic Disorders, Shanghai Mental Health Center Shanghai Jiaotong University School of Medicine Shanghai China; ^3^ Co‐innovation Center of Neuroregeneration Nantong University Nantong China

Risk of Schizophrenia originates from neurodevelopmental deficits induced by either genetic factors or environmental stressors.^1^ The clinical symptoms often appear after adolescence period, which can be predicted by abnormal pattern of brain activities prior to the first psychotic episode.^2^ Psychological intervention, pharmacological agents, behavioral therapies, and Invasive brain stimulation (eg, DBS) act as potential tools to reduce the schizophrenia development for population at high risk.^3^, ^4^, ^5^

In recent decade, noninvasive brain stimulation has been employed as potential treatments against cognitive deficits and negative symptoms in schizophrenia patients.^6^ Yet it is unclear if noninvasive brain stimulation could prevent schizophrenia development and progression as well. In a recent study by Hadar et al published on *Molecular Psychiatry*, the authors set out to demonstrate the possibility of adolescent period brain stimulation‐based prevention of schizophrenia development.^7^

The authors created the rat model of schizophrenia with maternal immune activation (MIA), which exhibits prepulse inhibition (PPI) deficits (a cross‐species behavioral parameter for sensorimotor gating), abnormal discrimination reversal (DR; for excessive switching), enhanced amphetamine‐induced activity (AIA; for enhanced dopaminergic transmission), and enlarged lateral ventricle (LV) volumes at adulthood. Considering the importance of prefrontal cortex (PFC) in cognition integrity and feasibility of clinical translation, the authors treated the PFC in adolescent MIA rats with transcranial direct current stimulation (tDCS; twice daily, 20 minutes, 13 days). The authors found that anodal (leading to excitation) but not cathodal/sham stimulation could prevent the development of reduced PPI, fast DR, enhanced AIA, and enlarged LV volume at adulthood. This suggested that tDCS stimulation of prefrontal cortex could prevent development of schizophrenia like behaviors and neuropathological changes in the offspring animals at high risk.

Interestingly, tDCS treatment did not reverse all behavioral changes in MIA animals, such as the anhedonia‐like behaviors, reduced social behaviors, and reduced parvalbumin (PV)‐expressing interneurons in mPFC. This suggested that either a stronger stimulation is required for more significant changes, or that PFC stimulation fails to target all neural circuits with pathological changes (eg, deeper limbic areas). It will also be interesting to see whether continuous treatment at adulthood could have additional effects on anhedonia and reduced sociality.

The study brings many questions to answer with future investigation. First, what is the mechanism underlying anodal tDCS benefits? It is supposed that anodal stimulation leads to depolarization of cortical neurons and enhance the excitability of the local region.^8^ It is yet unknown how the brain network is modulated following single or repeated tDCS sessions, and the depth of direct/indirect tDCS effect. Neurophysiological studies with multichannel recording of neuronal activities, or histological mapping with neuronal activity biomarkers (eg, cFos/Arc) would offer such evidences, which might explain the effectiveness of tDCS on some but not all behavioral phenotypes. It will also be helpful to measure the monoamine and neurotrophic factor release levels at multiple sites of the brain following tDCS treatment sessions.^9^

Second, what is the best time window for this tDCS intervention and how long should it be performed? Is it possible to start treatment earlier than adolescence, and without disruption of normal region functions? Is prolonged treatment better than a fix period? These are important questions to answer for safety cautions before clinical translation. Notably, the anodal stimulation over healthy developing animals actually brought certain behavioral defects in later period; it will be necessary to identify very reliable biomarkers prior to start the intervention.

Third, in clinical patients, risk population for schizophrenia is identified with varied early symptoms, associating with abnormal brain activities at distinct neural circuits.^2^ Whether targeting PFC results in benefits for all subtype of schizophrenia patients, or individualized treatment should be conducted for schizophrenia prevention is to be elucidated. This will require more preclinical studies and clinical trials to verify different possibilities.

In sum, the study for the first time demonstrated the possibility to prevent schizophrenia development with brain stimulation, for cohorts at high risk. The abnormal brain activity pattern has been recognized as potential biomarkers for upcoming psychosis, and targeting such changes with pharmacological or physical approaches might be efficient in early intervention. Other brain stimulation approaches (eg, transcranial magnetic stimulation, TMS) might offer additional tools for schizophrenia prevention.

## CONFLICT OF INTEREST

The authors declare no conflict of interest.
